# The Effect of the Molecular Weight of Polyvinylpyrrolidone and the Model Drug on Laser-Induced In Situ Amorphization

**DOI:** 10.3390/molecules26134035

**Published:** 2021-07-01

**Authors:** Nele-Johanna Hempel, Padryk Merkl, Matthias Manne Knopp, Ragna Berthelsen, Alexandra Teleki, Anders Kragh Hansen, Georgios A. Sotiriou, Korbinian Löbmann

**Affiliations:** 1 Department of Pharmacy, University of Copenhagen, 2100 Copenhagen, Denmark; nele.hempel@sund.ku.dk (N.-J.H.); ragna.berthelsen@sund.ku.dk (R.B.); 2 Department of Microbiology, Tumor and Cell Biology, Karolinska Institute, 17177 Stockholm, Sweden; padryk.merkl@ki.se (P.M.); georgios.sotiriou@ki.se (G.A.S.); 3 Bioneer:FARMA, Department of Pharmacy, University of Copenhagen, 2100 Copenhagen, Denmark; mmk@bioneer.dk; 4 Science for Life Laboratory, Department of Pharmacy, Uppsala University, 75123 Uppsala, Sweden; alexandra.teleki@scilifelab.uu.se; 5 Department of Photonics Engineering, Technical University of Denmark, 4000 Roskilde, Denmark; ankrh@fotonik.dtu.dk

**Keywords:** in situ amorphization, near-IR laser radiation, amorphous solid dispersion, plasmonic photothermal nanoparticles, dissolution kinetics

## Abstract

Laser radiation has been shown to be a promising approach for in situ amorphization, i.e., drug amorphization inside the final dosage form. Upon exposure to laser radiation, elevated temperatures in the compacts are obtained. At temperatures above the glass transition temperature (*T*_g_) of the polymer, the drug dissolves into the mobile polymer. Hence, the dissolution kinetics are dependent on the viscosity of the polymer, indirectly determined by the molecular weight (*M*_w_) of the polymer, the solubility of the drug in the polymer, the particle size of the drug and the molecular size of the drug. Using compacts containing 30 wt% of the drug celecoxib (CCX), 69.25 wt% of three different *M*_w_ of polyvinylpyrrolidone (PVP: PVP12, PVP17 or PVP25), 0.25 wt% plasmonic nanoaggregates (PNs) and 0.5 wt% lubricant, the effect of the polymer *M*_w_ on the dissolution kinetics upon exposure to laser radiation was investigated. Furthermore, the effect of the model drug on the dissolution kinetics was investigated using compacts containing 30 wt% of three different drugs (CCX, indomethacin (IND) and naproxen (NAP)), 69.25 wt% PVP12, 0.25 wt% PN and 0.5 wt% lubricant. In perfect correlation to the Noyes–Whitney equation, this study showed that the use of PVP with the lowest viscosity, i.e., the lowest *M*_w_ (here PVP12), led to the fastest rate of amorphization compared to PVP17 and PVP25. Furthermore, NAP showed the fastest rate of amorphization, followed by IND and CCX in PVP12 due to its high solubility and small molecular size.

## 1. Introduction

In situ amorphization describes the amorphization of a crystalline drug inside the final dosage form, such as a compact [1]. The amorphization can take place right after the manufacturing of the compact or directly before administration. In situ amorphization has been shown upon immersion in water, upon exposure to convectional heating and upon exposure to microwave and laser radiation [2,3,4,5]. Only using the latter two methods, complete amorphization has been obtained [4,5,6]. In order for a compact to absorb electromagnetic radiation, such as microwave or laser radiation, enabling excipients are necessary inside the compact [7,8,9].

In a recent study, photothermal silver plasmonic nanoaggregates (PNs) [10] were shown to be a promising enabling excipient for laser-induced in situ amorphization and were found to be non-toxic to Caco-2 cells at relevant doses. However, further studies are still needed to fully evaluate the toxicological profile of silver PNs associated with their oral administration [4]. Low amounts (≤0.25 wt%) of PNs, which absorb light in the near-infrared region, have been shown to be sufficient to enable laser-induced in situ amorphization [4]. The approach was successfully utilized in a proof-of-concept study, in which complete amorphization of 30 wt% and 50 wt% of the model drug celecoxib (CCX) in the polymer polyvinylpyrrolidone (PVP12) was obtained upon exposure to laser radiation. The study showed that a faster rate of amorphization was obtained for compacts containing higher amounts of PNs (0.25 wt% vs. 0.1 wt%), as well as for compacts exposed to laser radiation at higher laser intensities (for example, 1.31 W/cm^2^ vs. 0.93 W/cm^2^). However, the study was limited to the drug CCX and PVP12 [4].

PNs have a unique surface property, called localized surface plasmon, which allows the PNs to absorb radiation around their resonance frequency [8,11]. In this study, the PNs have been selected to absorb laser radiation in the near-infrared region [12,13]. Upon exposure to laser radiation, the PNs generate heat, which leads to a temperature increase inside the compact. Once temperatures above the glass transition temperature (*T*_g_) of the polymer are reached, the drug readily dissolves into the mobile polymer and forms an amorphous solid dispersion (ASD) [14,15,16]. In previous studies, it was suggested that the rate of drug dissolution into the polymer only becomes significant when the compact temperature is above a certain temperature, labelled the temperature of the “onset” of the drug dissolution (*T*_Onset_) [4,14,15]. As significant drug dissolution only occurs, when the polymer is mobile, i.e., at temperatures above the *T*_g_ of the polymer (*T*_Onset_ > *T*_g_), *T*_Onset_ was defined as the temperature threshold, which needs to be surpassed upon exposure to laser radiation to achieve (measurable) in situ amorphization. Drug dissolution is kinetically limited at temperatures below the *T*_g_ of the polymer due to a high viscosity [14,17,18,19]. Furthermore, as in situ amorphization follows a dissolution process, which is a time- and temperature-dependent process [20,21], a certain temperature needs to be surpassed in the relatively short time-frame of laser exposure in order to obtain complete amorphization. With increasing drug dissolution into the mobile polymer, the viscosity of the polymer increases [22], which increases the temperature necessary to continue the dissolution process at the same dissolution speed and/or within the given time-frame. The temperature to (theoretically) obtain complete amorphization is labelled as the minimum temperature, *T*_Min_, where *T*_Min_ > *T*_Onset_. Both *T*_Min_ and *T*_Onset_ can be determined by thermal analysis (differential scanning calorimetry, DSC) for each drug–polymer combination and analyzing the dissolution endotherm of the obtained thermograms. The three temperatures, *T*_g_, *T*_Onset_ and *T*_Min_, for the different compact compositions have been used to pre-assess which temperatures are necessary during exposure to laser radiation to achieve in situ amorphization.

The aim of the present study was two-fold: (i) to investigate the effect of the molecular weight (*M*_w_) of the polymer PVP on the dissolution of the drug into the polymer and (ii) to investigate the effect of the model drug on the dissolution into the polymer PVP12.

For (i), 30 wt% of the model drug CCX was used together with PVP in different *M*_w_ grades, namely PVP12, PVP17 and PVP25. As the complete amorphization of CCX in PVP12 has been shown previously [4], it remains to be investigated how the rate and degree of amorphization of laser-induced in situ amorphization are affected using a higher *M*_w_ of PVP, i.e., the same polymer with a higher viscosity due to a higher *M*_w_. It is suggested based on studies investigating microwave-induced in situ amorphization that a higher viscosity due to a higher *M*_w_ will result in a slower rate and, therefore, an overall lower degree of amorphization upon exposure to radiation [22,23]. As the rate of amorphization is also dependent on the ‘drug in polymer’ solubility, it is important to mention that the solubility of CCX in PVP is independent of the *M*_w_ of PVP, i.e., CCX has the same solubility in the polymers PVP12, PVP17 and PVP25 [24]. The predicted solubility of CCX in PVP12 was previously reported at 41 wt% at 20 °C (confidence interval: 30–48 wt%) [25]. This allows the investigation of the effect of the *M*_w_ of the polymer independently of the drug solubility in the polymer. The chosen drug load of 30 wt% is below the predicted solubility limit at room temperature; hence, all drugs should be able to dissolve into PVP under the experimental conditions.

For (ii), PVP12 was used with three different drugs, namely CCX, indomethacin (IND) and naproxen (NAP). All three drugs show solubility in PVP/PVP12 in the temperature range of the experimental conditions (i.e., from room temperature to the maximum temperature obtained during exposure to laser radiation). Furthermore, the three drugs are different in their glass-forming ability, i.e., NAP is a poor (class I), CCX a medium (class II) and IND a good glass-former (class III) [26] and have been previously used in the formation of ASD. Considering the drug molecules, it is not only the drug solubility in the polymer that has an effect on the dissolution rate, i.e., the rate of amorphization upon exposure to laser radiation, but also the molecular size of the drug molecule [20,21]. The molecular size of a drug molecule can be estimated in 2D by its minimum and maximum projected diameter. NAP has diameters of 1.1 and 0.5 nm. The diameters of IND are 1.2 and 0.8 nm, whilst CCX has diameters of 1.1 and 1.0 nm. Hence, the molecular size of the drug molecules chosen for this study increase in the following order: NAP < IND < CCX. It is suggested, according to the Noyes–Whitney equation [20], that a smaller drug molecule will show enhanced dissolution kinetics, i.e., a higher rate of amorphization.

## 2. Results and Discussion

### 2.1. The Effect of the Molecular Weight of Polyvinylpyrrolidone on In Situ Amorphization

Using PVP12, PVP17 and PVP25, the influence of the *M*_w_ of PVP, i.e., the viscosity of the polymer, on the in situ amorphization of the drug CCX was investigated. It is commonly known that the viscosity and the *T*_g_ increase with the *M*_w_ of the polymer [27], which results in the following order for the increase in viscosity of PVP: PVP12 < PVP17 < PVP25. The viscosity of the polymer affects the dissolution kinetics of the drug into the mobile polymer, according to the diffusion coefficient of the Noyes–Whitney equation [20]. Furthermore, the increase in the *T*_g_ with increasing *M*_w_ of the polymer is suggested to result in an increase in *T*_Onset_ and *T*_Min_ of the compacts.

#### 2.1.1. The Determination of the Temperature Thresholds, T_Onset_ and T_Min_

Prior to exposure to laser radiation, the *T*_g_ of the polymers, as well as the *T*_Onset_ and *T*_Min_, were determined by DSC. The results are shown in Table 1. As can be seen, a higher *M*_w_ of the polymer resulted in a higher *T*_g_ of the polymer, which resulted in a higher *T*_Onset_ and *T*_Min_ for compacts containing the respective polymer. The temperature thresholds, *T*_Onset_ and *T*_Min_, were used as a pre-assessment to determine which temperatures were necessary to achieve (measurable) and complete in situ amorphization, respectively. Hence, a higher *M*_w_ of the polymer resulted in overall higher temperatures necessary to initiate the drug dissolution into the polymer and to obtain a fully amorphous ASD.

From Table 1, it can be seen that *T*_Onset_ is a temperature between *T*_g_1 and *T*_g_2, i.e., a temperature where the polymer is mobile enough for the drug to dissolve in. The significant decrease in viscosity and thereby increase in mobility has previously been reported at approximately *T*_g_ + 15– 25 °C (Note: *T*_g_ determined by differential scanning calorimetry; here *T*_g_1) [4,14,17]. As PVP contains water, which evaporates during exposure to laser radiation, the *T*_g_ will increase during exposure to laser radiation and will be between *T*_g_1 and *T*_g_2 during the exposure (not all water evaporates). PVP12, PVP17 and PVP25 contained 5.3 ± 0.3 wt%, 9.5 ± 0.3 wt% and 7.8 ± 0.1 wt% water, respectively (mean ± SD, *n* = 3). *T*_min_ is the minimum temperature necessary to obtain a fully amorphous ASD during exposure to laser radiation. *T*_min_ is always at higher temperatures than *T*_Onset_ and was found to be above *T*_g_2 for CCX.

Based on the results of the thermal analysis, it was suggested that CCX would amorphize at a lower temperature and/or faster rate at the same temperature in compacts containing PVP12, followed by PVP17 and PVP25.

#### 2.1.2. The Rate of Amorphization upon Exposure to Laser Radiation

**Figure 1:**
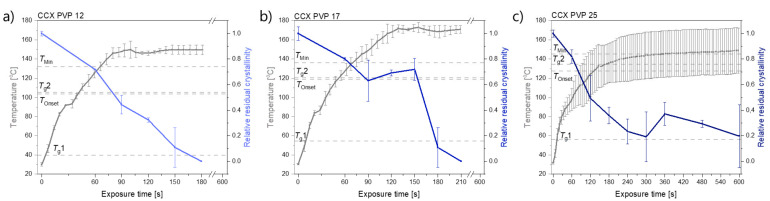
Temperature [°C] (left *y*-axis) and relative residual crystallinity (right *y*-axis, determined by DSC analysis) plotted as a function of exposure time [s]. All compacts were exposed at a laser intensity of 1.52 W/cm^2^. (**a**) Compacts containing CCX and PVP12 (light blue), (**b**) Compacts containing CCX and PVP17 (blue), (**c**) Compacts containing CCX and PVP25 (dark blue). Mean ± SD (*n* = 3) for the left *y*-axis and mean ± SD (*n* = 2) for the right *y*-axis. The horizontal dashed lines indicate the temperatures, *T*_g_1, *T*_g_2, *T*_Onset_ and *T*_Min_, as seen in Table 1.

**Figure 2:**
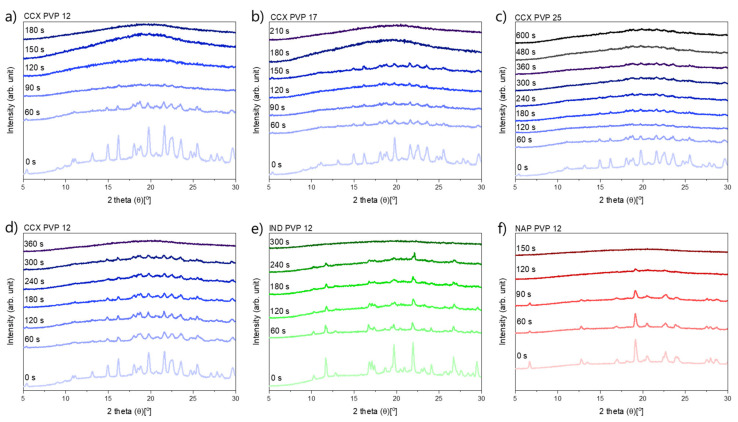
XRP-diffractograms at various exposure times. (**a**–**c**) exposed at a laser intensity of 1.52 W/cm^2^, (**a**) CCX PVP12, (**b**) CCX PVP17, (**c**) CCX PVP25, (**d**–**f**) exposed at a lower laser intensity of 1.02 W/cm^2^, (**d**) CCX PVP12, (**e**) IND PVP12, (**f**) NAP PVP12.

In Figure 1, it can be seen that compacts containing CCX and PVP12 showed a faster rate of amorphization upon exposure to laser radiation than compacts containing PVP17. Compacts containing CCX and PVP12 became fully amorphous after 180 s of exposure to laser radiation, whilst compacts containing CCX and PVP17 required 210 s to become fully amorphous (Figure 1a,b). The relative residual crystallinity was determined by DSC analysis of the compacts before and after exposure to laser radiation. Complete amorphization was confirmed by XRPD (see Figure 2a–c).

Compacts containing CCX and PVP25 did not become fully amorphous. The temperatures obtained did not reach *T*_Min_ in any of the three investigated compacts to allow for complete amorphization, which can also be seen in the deviation of the results obtained for the relative residual crystallinity (Figure 1c). It is suggested that higher temperatures and/or longer exposure times would be necessary to achieve complete amorphization for compacts containing CCX and PVP25.

For compacts containing CCX and PVP12 or PVP17, the temperatures reached during exposure to laser radiation were above *T*_Min_, which means complete amorphization was possible at the obtained temperatures if sufficient time at this temperature was ensured. As the temperature thresholds, *T*_Onset_ and *T*_Min_, were lower for compacts containing CCX and PVP12 compared to compacts containing PVP17, a faster rate of amorphization was obtained, even though higher maximum temperatures were obtained for compacts containing CCX and PVP17 (149.6 ± 4.9 °C (CCX/PVP12 compacts) vs. 171.0 ± 3.8 °C (CCX/PVP17 compacts); mean ± SD (*n* = 3)). In both cases, the temperature increase was found to be similar in the first 90 sec of exposure to laser radiation approximately until *T*_Min_ was reached for compacts containing CCX and PVP12. It can be concluded that a polymer with a lower *M*_w,_ and therefore a lower viscosity, resulted in an overall faster rate of amorphization compared to a higher *M*_w_ of the same polymer kind. These results are in line with previous results reported for microwave-induced in situ amorphization [22,23]. The results of this study also show that higher *M*_w_ of a polymer, e.g., PVP17, can be used for laser-induced in situ amorphization. This was not found for microwave-induced in situ amorphization, where PVP17 was not suitable to amorphize the same CCX-PVP17 combination. In a previous study, the compacts used for microwave-induced in situ amorphization contained 30 wt% CCX and PVP17 and were conditioned at high relative humidity to allow for water sorption. The sorbed water acted as a dielectric heating source and plasticizer, i.e., lowering the *T*_g_ of PVP17. During exposure of the compacts to microwave radiation, the sorbed water evaporated fast, which increased the *T*_g_ to a temperature that was not reachable during microwave irradiation, i.e., the amorphization came to a halt [23]. With the use of laser radiation instead of microwave radiation, polymers with a higher *M*_w_ and higher *T*_g_ become available for in situ amorphization, as it was shown that higher temperatures could be obtained.

### 2.2. The Influence of the Model Drug on In Situ Amorphization

Using PVP12, the influence of the model drug on the in situ amorphization upon exposure to laser radiation was investigated. For this, CCX, IND and NAP were utilized.

#### 2.2.1. Solubility of Celecoxib, Indomethacin and Naproxen in Polyvinylpyrrolidone

**Figure 3:**
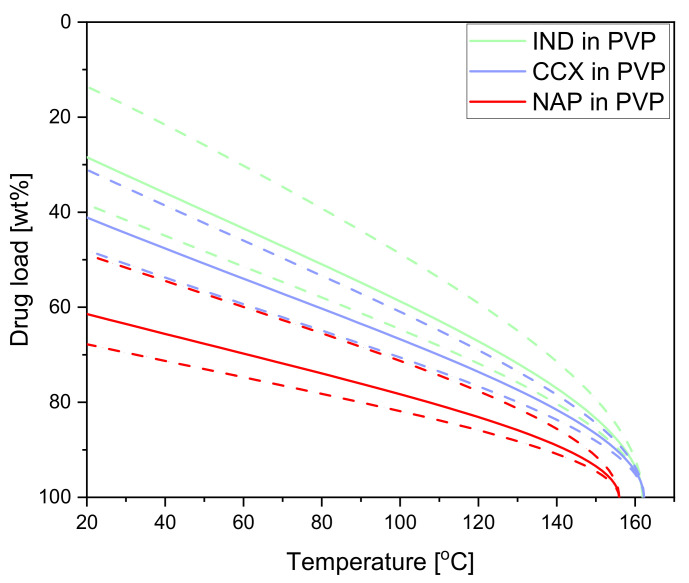
(Predicted) drug solubility curves for IND in PVP (green), CCX in PVP (blue) and NAP in PVP (red). Dashed lines indicate the confidence interval. The data for IND and CCX in PVP was taken from [28].

All model drugs show significant (predicted) solubility in PVP12 at room temperatures and the temperatures obtained during exposure to laser radiation. CCX, as mentioned above, has a predicted solubility of 41 wt% (confidence interval: 30–48 wt%) at 20 °C in PVP12. IND has a lower predicted solubility of 28 wt% (confidence interval: 14–38 wt%) at 20 °C [28]. The predicted solubility of NAP in PVP12 was determined to be at 61 wt% (confidence interval: 48–67 wt%) at 20 °C (see Figure 3). Hence, NAP has the highest predicted solubility in PVP12 at 20 °C, followed by CCX and IND. In the case of CCX and NAP, the chosen drug load of 30 wt% is below the predicted solubility limit at room temperature. For the drug IND, the chosen drug load of 30 wt% is in the confidence interval of the predicted solubility at room temperature. As the solubility increases with increasing temperature, a drug load of 30 wt% IND should be able to dissolve in PVP12 during the study. For example, at the maximum compact temperature (129.9 ± 2.4 °C), 64–76 wt% IND may, in theory, be soluble in PVP12.

#### 2.2.2. The Determination of the Temperature Thresholds, T_Onset_ and T_Min_

To pre-assess which temperatures (achieved upon exposure to laser radiation) were necessary to obtain complete in situ amorphization, the temperature thresholds of IND and NAP in PVP12 were also determined by DSC. As seen in Table 1, compacts containing CCX, IND, or NAP and PVP12 had different temperature thresholds. Even though the temperatures for the polymer *T*_g_1 and *T*_g_2 were identical, *T*_Onset_ and *T*_Min_ were different for each drug in PVP12. *T*_Onset_ and *T*_Min_ decreased in the following drug order: CCX > IND > NAP. Based on these results, it was suggested that compacts containing NAP and PVP12 would become fully amorphous at lower temperatures (and therefore following shorter exposure times) compared to compacts containing IND or CCX.

#### 2.2.3. The Rate of Amorphization upon Exposure to Laser Radiation

**Figure 4:**
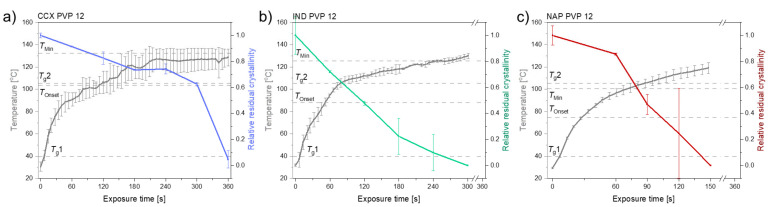
The temperature (°C) (left *y*-axis) and relative residual crystallinity (right *y*-axis, determined by DSC analysis) plotted as a function of exposure time [s]. All compacts were exposed at a laser intensity of 1.02 W/cm^2^. (**a**) Compacts containing CCX and PVP12 (light blue), (**b**) Compacts containing IND and PVP12 (light green), (**c**) Compacts containing NAP and PVP12 (red). Mean ± SD (*n* = 3) for the left *y*-axis and mean ± SD (*n* = 2) for the right *y*-axis. The horizontal dashed lines indicate the temperatures, *T*_g_1, *T*_g_2, *T*_Onset_ and *T*_Min_, as seen in Table 1.

Figure 4 shows the different compact temperatures and rates of amorphization obtained for compacts containing CCX, IND or NAP and PVP12 upon exposure to laser radiation.

It can be seen from Figure 4 that compacts containing NAP and PVP12 showed a faster rate of amorphization upon exposure to laser radiation than compacts containing IND or CCX. Compacts containing NAP and PVP12 became fully amorphous after 150 s, whilst compacts containing IND or CCX and PVP12 were fully amorphous after 300 s and 360 s, respectively (Figure 4). The complete amorphization of all compacts was confirmed by XRPD (see Figure 2d–f). For compacts containing NAP or IND, the compact temperatures reached were above *T*_Min_, and complete amorphization was obtained during exposure to laser radiation. For compacts containing CCX and PVP12, the compact temperatures reached during exposure to laser radiation were just below *T*_Min_. However, due to the differences in water content between the determination of *T*_Min_ using DSC and the compact exposed to laser radiation, it is suggested that the theoretically calculated value *T*_Min_ might be slightly overestimated, i.e., as shown above, complete amorphization was possible at temperatures just below the determined *T*_Min_. As the temperature thresholds, *T*_Onset_ and *T*_Min_, were the lowest for compacts containing NAP as compared to compacts containing IND or CCX, a faster rate of amorphization was indeed obtained as suggested.

As the same polymer, and thereby the same viscosity, was used for this series of experiments, the rate of amorphization of the three model drugs into PVP 12 was only expected to be affected by the characteristics of the drugs. That means the dissolution of the three model drugs was affected by their solubility in the PVP12, their molecular sizes and their particle size, which affects the surface area available for dissolution, as described by the Noyes–Whitney equation (first-order dissolution kinetic) [20]. Due to the preparation method of the mixtures, similar particle sizes of the drugs (and PVP) can be expected, i.e., differences in the amorphization rate origin from the different solubilities in PVP12 and different molecular sizes of the drugs. However, the amorphization rate does not increase in the same order as seen for the drug–polymer solubilities, i.e., it is suggested that the molecular size of the drug dictated the amorphization rate in this study. Furthermore, the temperature thresholds determined follow the same order as the molecular size of the drug, e.g., NAP as the smallest molecule has the lowest temperature threshold, which also strongly indicates that the molecular size of the drug determined the amorphization rate.

It can be concluded that the use of compacts containing a drug with a moderate to high solubility in the polymer, low temperature thresholds and small molecular size resulted in an overall faster rate of amorphization compared to drugs with a lower solubility in the polymer, higher temperature thresholds and larger molecule sizes.

### 2.3. Chemical Stability upon In Situ Amorphization

As increased temperatures are obtained upon exposure to laser radiation, compacts exposed to the longest exposure times were tested for drug degradation using high-performance liquid chromatography (HPLC). No degradation was detected in any of the compacts (data not shown). As high temperatures are obtained during exposure to laser radiation, the thermal stability of the drugs (and polymers) is of great importance, i.e., possible degradation could alter the bioavailability and biological activity of certain thermosensitive drugs.

## 3. Materials and Methods

### 3.1. Materials

Celecoxib (CCX, *M*_w_ = 381.4 g/mol), indomethacin (IND, *M*_w_ = 357.8 g/mol), naproxen (NAP, *M*_w_ = 230.3 g/mol) and magnesium stearate (*M*_w_ = 591.3 g/mol) were purchased from Fagron Nordic A/S (Copenhagen, Denmark). Kollidon^®^ 12 PF (polyvinylpyrrolidone (PVP), PVP12, *M*_w_ ~2500 g/mol), Kollidon^®^ 17 PF (PVP17, *M*_w_ ~9000 g/mol) and Kollidon^®^ 25 (PVP25, *M*_w_ ~24,000 g/mol) were a kind gift from BASF (Ludwigshafen, Germany).

Silver acetate (99.8% anhydrous) was purchased from Alfa Aesar (Kandel, Germany). Hexamethyldisiloxane (≥98%), acetonitrile (99.8% anhydrous) and 2-ethylhexanoic acid (99%) were purchased from Sigma-Aldrich (Stockholm, Sweden). The oxygen gas (flow: 5.0 L/min) for the flame-spray-pyrolysis synthesis was from Strandmøllen (Ljungby, Sweden).

Ethanol (>99.7%, HPLC grade) was purchased from VWR International (Leuven, Belgium). Acetonitrile (HPLC grade) and trifluoroacetic acid were purchased from Sigma-Aldrich (Søborg, Denmark). Purified water used for the mobile phase in the HPLC experiments was prepared using a MilliQ water system from LabWater (Los Angeles, CA, USA). All chemicals were used as received.

### 3.2. Plasmonic Nanoaggregate Synthesis

Silver PNs were obtained by flame-spray pyrolysis with a final composition of 98 wt% silver and 2 wt% silicon dioxide (spacer) [29], as introduced by Sotiriou et al. 2010 and 2011 [10,30] and Merkl et al. 2021 [12]. A detailed description of the procedure can be found in Hempel et al. (2021) [4].

### 3.3. Compact Preparation

Physical mixtures containing 30 wt% drug (CCX, IND or NAP), 69.25 wt% polymer (PVP12, PVP17 or PVP25), 0.5 wt% magnesium stearate (lubricant) and 0.25 wt% PN were obtained using mortar and pestle. A total of 50 ± 2 mg of each physical mixture was compressed into flat-faced compacts (diameter: 6 mm) using an instrumented single-punch GTP-1 tablet machine from Gamlen Instruments (Nottingham, UK). The tablet machine was equipped with a 500 kg load cell (CT6-500-022) and used at a compaction pressure of 160 MPa.

### 3.4. In Situ Amorphization

In situ amorphization was induced using laser radiation at a wavelength of 805 nm (Near-infrared). The compacts were exposed to laser radiation for various exposure times, as indicated in Table 2. The maximum exposure time was set to 600 s. The laser was a fiber-coupled laser diode, controlled using a Newport Laser Diode Controller Model 6000 (Mölndal, Sweden). The current to the laser diode was 4000 mA, resulting in a laser power of 3321 mW ex fiber. No collimation lens was used at the fiber output, so the beam was diverging upon exiting the fiber, allowing for easy adjustment of laser intensity on the compact by varying the distance. The distance from the fiber tip to the bottom of the compact was either set to 6.75 cm or 8.25 cm, which corresponds to a laser intensity of 1.52 or 1.02 W/cm^2^, respectively. The two different laser intensities were used as at the high laser intensity, and the amorphization of compacts containing NAP and PVP12 was very fast, and the difference between IND and CCX compacts could not be determined, i.e., it was necessary to lower the laser intensity to investigate the influence of the drug in Section 2.2. At the low laser intensity, very long exposure times were necessary for compacts containing PVP17 and PVP25, exceeding 600 s. Each compact was located on a cover glass slide and covered by another cover glass slide.

During in situ amorphization, the surface temperature of the cover glass slide covering the compacts was measured using an infrared thermocamera testo 871 from Testo SE & CO. KGaA (Lenzkirch, Germany). Using the thermography app, thermal images were approximately recorded every sixth sec and analyzed afterwards using the testo IRSoft Software (version 4.5). The thermal images were exported as .xlsx files and analyzed for the maximum temperature at each time point using Matlab R2019a (version 9.6.0.1335978) from Mathworks (Natick, MA, USA). Each experiment was conducted as a triplicate (*n* = 3).

### 3.5. Qualitative Analysis of Crystallinity by Solid-State Analysis

Solid-state analysis of the pure substances and the compacts before- and after exposure to laser radiation was performed using X-ray powder diffraction (XRPD). Prior to the analysis, the compacts were powdered using a mortar and pestle. The analysis was performed using an X’Pert Pro diffractometer from PANalytical (Eindhoven, The Netherlands) with Cu Kα radiation (λ = 1.54187 Å). The diffractograms were recorded from 5 to 30° 2θ at 45 kV and 40 mA. The diffractograms were analyzed with the X’Pert HighScore Plus software (version 2.2.4.) from PANalytial (Eindhoven, The Netherlands).

### 3.6. Thermal Analysis and Quantification of Crystallinity

Thermal analysis was performed using a Q2000 DSC from TA Instruments (New Castle, DE, USA). All experiments were conducted under a nitrogen gas purge with a flow of 50 mL/min. The data was analyzed using the TRIOS software (version 5.1.1) from TA Instruments. The DSC cell constant was calibrated using indium.

#### 3.6.1. Quantification of the Residual Drug Crystallinity

To quantify the residual crystallinity in compacts exposed to laser radiation, calibration curves were established for the drugs CCX, IND and NAP in PVP (PVP12, PVP17 and PVP25 for CCX and PVP12 for IND and NAP). Using a mortar and pestle, 100 mg physical mixtures containing 10–30 wt% drug (in 5 wt% increments) and 90–70 wt% PVP (12, 17 or 25) were obtained. Of each mixture, 3–5 mg was weighed into a Tzero aluminum pan and sealed with a perforated hermetic lid. A modulated DSC (mDSC) run was performed with a heating rate of 3 °C/min from 20 °C to 180 °C. The modulation had an amplitude of 1 °C/50 s. The dissolution enthalpy was determined in the total heat flow using the TA Instruments TRIOS software (version 5.1.1). Each calibration point was determined as a duplicate (*n* = 2). As the water evaporated upon heating, the sample mass was corrected by the water content of the pure polymer (see Section 3.7). The calibration curve was plotted and fitted with a polynomial function of the second degree with an intercept of 0.

Compacts before -, and after exposure to laser radiation were carefully mortared, and 2–5 mg of the powdered compacts were weighted into Tzero aluminum pans and sealed with a perforated hermetic lid. The dissolution enthalpy was determined using a modulated DSC (mDSC) run as described above. Each experiment was conducted as a duplicate (*n* = 2). The sample mass was also corrected by the water content (see Section 3.7). Using the established calibration curve, the residual crystallinity was calculated from the determined dissolution enthalpy.

#### 3.6.2. Determination of the (Predicted) Solubility of Naproxen in Polyvinylpyrrolidone

For compacts containing NAP and PVP12, the drug-polymer solubility was determined. For this, 100 mg physical mixtures were obtained by mortar and pestle with 70–90 wt% NAP in 5 wt% increments. Afterwards, 3–5 mg of each mixture, and additionally the pure NAP, were weighed into Tzero aluminum pans and closed with a perforated hermetic lid. A temperature ramp of 1 °C/min to 180 °C was applied. Each experiment was conducted as a duplicate (*n* = 2). The onset of melting was determined. Using the Flory-Huggins approach [31,32], the solubility of the NAP in the PVP12 was calculated from the onset of the dissolution endotherm. Details of the method can be found in Knopp et al. (2015) [33].

#### 3.6.3. Glass Transition Temperatures of the Polymers

The *T*_g_ of the polymers was also determined by thermal analysis. For each polymer, PVP12, PVP17 and PVP25, 3–5 mg was weighed into Tzero aluminum pans with hermetically sealing lids. In the case of determination of the water-free *T*_g_ (*T*_g_2), the lid was perforated to allow the bulk water to evaporate. Each sample was first heated to 120 °C, followed by an isothermal period of 10 min before equilibrating to 20 °C. Subsequently, the sample was subjected to a modulated DSC (mDSC) run from 20 to 180 °C at a heating rate of 3 °C/min with an amplitude of 1 °C/50 s. For the determination of the *T*_g_ with bulk-water (*T*_g_1), the sample was heated from 20 to 120 °C using the above mDSC settings. The *T*_g_ was determined as the midpoint of the step change. Each experiment was conducted as a duplicate (*n* = 2).

#### 3.6.4. Determination of the Temperature Thresholds, T_Onset_ and T_Min_

Using a mortar and pestle, 100 mg physical mixtures containing 30 wt% drug, CCX, IND or NAP and 70 wt% PVP (12, 17, 25, see Table 2) were obtained. Of each mixture, 3–5 mg was weighed into a Tzero aluminum pan and sealed with a perforated hermetic lid. A heating rate of 0.5 °C/min, 1 °C/min, 2 °C/min, 3 °C/min, 5 °C/min or 10 °C/min was applied from 20 to 180 °C. For each experiment, the on- and endset of the dissolution endotherm were determined. The values obtained for the different heating rates were extrapolated to a heating rate of 0 °C/min, which represents *T*_Onset_ (for the onset of the dissolution at a heating rate of 0 °C/min) and *T*_Min_ (for the endset of dissolution at a heating rate of 0 °C/min).

### 3.7. Determination of the Water Content

The water content of the polymers, PVP12, PVP17 and PVP25, the physical mixtures, and the compacts after exposure to laser radiation was determined using a Discovery thermogravimetric analyzer (TGA) from TA instruments Inc. (New Castle, DE, USA). The experiments were conducted under a nitrogen gas purge of 25 mL/min, and the weight loss was determined using the TA Instruments TRIOS software (version 5.1.1).

The water content of each sample was determined by heating of 10 °C/min from ambient temperature to 170 °C. Each experiment for the pure compounds and the physical mixtures was conducted as a triplicate (*n* = 3) or duplicate (*n* = 2), respectively. The water content of the compacts exposed to laser radiation was determined for each exposure time and each compact composition as a single run (*n* = 1).

### 3.8. Determination of the Drug Molecule Size

The drug molecule size, i.e., the minimum and the maximum projected diameter, was determined using the Avogadro software, version 1.2.0. Avogadro is an open-source molecular builder and visualization tool: http://avogadro.cc/ (accessed on 5 February 2021).

### 3.9. Drug Degradation

HPLC was used to quantify the amount of CCX, IND and NAP in the compacts before and after exposure to laser radiation, as well as to determine any signs of drug degradation. The experiments were performed using a 1260 Infinity HPCL from Agilent Technologies, Inc. (Santa Clara, CA, USA) on a reverse-phase Luna 5U C18(2) 100 A column with a length of 150 mm and a diameter of 4.60 µm from Phenomenex Ltd. (Aschaffenburg, Germany). The HPLC analysis was performed at ambient temperature. The mobile phases were degassed before use (Note: trifluoroacetic acid was added after degassing of the purified water, as it would evaporate otherwise). Each sample was injected as a triplicate (*n* = 3).

The HPLC method for CCX was as follows: the mobile phase, purified water and ethanol, was eluted at a ratio of 3:7 (*v*/*v*) with a flow rate of 1 mL/min. A sample volume of 10 µL was injected, and UV-detection was performed at a wavelength of 251 nm. The elution of CCX occurred at a retention time of 2.6 min.

The applied HPLC method for IND was adapted from Palmelund et al. (2019) [34]. Acetonitrile and purified water with 0.05% trifluoroacetic acid were used as the mobile phase in a 1:1 (*v*/*v*) ratio. The mobile phase was eluted at a flow rate of 1 mL/min, and a sample volume of 10 µL was injected. UV detection was performed at a wavelength of 264 nm. The retention time was found to be at 10.9 min. The same HPLC method was applied for NAP, which was detected as a wavelength of 270 nm and a retention time of 5.5 min.

To determine the drug content of the physical mixtures and compacts after the respective longest exposure time to laser radiation, HPLC samples were prepared by dissolving the physical mixtures or the mortared compacts in 50.0 or 100.0 mL of the organic phase of the respective mobile phase, i.e., ethanol in the case of CCX or acetonitrile for IND and NAP. Subsequently, the solution was filtered using a nylon syringe filter Q-max^®^ RR 25 mm with a pore size of 0.45 µm from Frisenette Aps (Knebel, Danmark), and the samples were analyzed by HPLC. The sample mass weight was corrected by the water content of the compact or mixture (see Section 3.7).

## 4. Conclusions

This study showed that for using PVP with different *M*_w_, a lower *M*_w_ is advantageous for the laser-induced in situ amorphization as it led to a faster rate of amorphization. The lower viscosity of PVP12 compared to PVP17 and PVP25 allowed the drug CCX to dissolve faster into the polymer during exposure to laser radiation, resulting in shorter exposure times until complete amorphization was reached. It is suggested that the same trend can be seen for other drugs or polymers. Using the same polymer, PVP12, but three different soluble model drugs, NAP, IND, and CCX, it could be shown that NAP resulted in the fastest rate of amorphization. The smaller molecular size of NAP, the lower temperature thresholds and the high solubility of NAP in PVP12 led to a faster rate of amorphization compared to compacts containing IND or CCX and PVP12.

## Figures and Tables

**Table 1:** The determination of the compact temperature thresholds, i.e., *T*_Onset_ and *T*_Min_, i.e., the temperatures where the in situ amorphization starts and is completed, respectively. *T*_g_1 is the *T*_g_ of the bulk polymer containing water. *T*_g_2 is the *T*_g_ of the water-free polymer. *T*_g_1 and *T*_g_2 are given as the Mean ± SD (*n* = 2).

Compact Composition	*T*_Onset_ [°C]	*T*_Min_ [°C]	Polymer	*T*_g_1 (Bulk) [°C]	*T*_g_2 (Water-Free) [°C]
30 wt% CCX PVP12	103.6	132.2	PVP12	39.7 ± 0.2	105.3 ± 0.5
30 wt% CCX PVP17	119.0	136.2	PVP17	54.5 ± 2.9	120.6 ± 0.1
30 wt% CCX PVP25	127.8	145.4	PVP25	56.1 ± 0.6	134.5 ± 1.5
30 wt% IND PVP12	88.0	125.6	PVP12	39.7 ± 0.2	105.3 ± 0.5
30 wt% NAP PVP12	74.5	100.5	PVP12	39.7 ± 0.2	105.3 ± 0.5

**Table 2:** Exposure times of compacts to laser radiation and the solid state of the drug determined by XRPD; c: residual crystallinity; a: fully amorphous. The experiments for the upper three rows were performed at a laser intensity of 1.52 W/cm^2^. The experiments for the lower three rows were performed at a laser intensity of 1.02 W/cm^2^.

Compact Composition	60 s	90 s	120 s	150 s	180 s	210 s	240 s	300 s	360 s	480 s	600 s
CCX PVP12	c	c	c	c	a						
CCX PVP17	c	c	c	c	c	a					
CCX PVP25	c		c		c		c	c	c	c	c
CCX PVP12	c	c	c	c	c	c	c	c	a		
IND PVP12	c	c	c	c	c	c	c	a			
NAP PVP12	c	c	c	a							

## Data Availability

The raw data is available from the authors upon request.

## Sample Availability

Samples are not available.
